# Preparing Students to Care for Socially Excluded Patients: A Qualitative Study of Inclusion Health in Undergraduate Medical Education

**DOI:** 10.1111/tct.70277

**Published:** 2025-12-10

**Authors:** Gemma Ashwell, Amy M. Russell, Lindsey Pope, Andrea Williamson

**Affiliations:** ^1^ Faculty of Medicine and Health University of Leeds Leeds UK; ^2^ School of Health and Wellbeing University of Glasgow Glasgow UK

## Abstract

**Introduction:**

Extreme health inequities are experienced by Inclusion Health groups (including people experiencing homelessness, problem substance use, Gypsy, Roma and Traveller communities, vulnerable migrants, sex workers, people in contact with the justice system and victims of modern slavery). There is evidence that undergraduate medical education is failing to prepare students to work effectively with these socially excluded groups. This research explores challenges and opportunities in teaching Inclusion Health to medical students.

**Methods:**

Twenty‐three educators involved in teaching Inclusion Health at medical schools in the United Kingdom and Ireland were recruited purposively through known contacts and snowball sampling. Semistructured interviews were conducted, and the interview transcripts were analysed using reflexive thematic analysis. An inductive approach was taken, and the analysis was underpinned by a critical realist ontology.

**Results:**

Five distinct themes were identified from the data:

1. ‘My goodness me, it's difficult to get that stuff in’; creating space for Inclusion Health in undergraduate curricula
2. ‘It's the human‐to‐human connection’; the importance of meaningful contact with people with lived experience
3. The impact of the hidden curriculum
4. ‘Assessment is the biggest hurdle’
5. Inclusion Health as a core competency for clinical practice

**Conclusion:**

Inclusion Health groups, who face intersecting forms of exclusion such as poverty, violence and trauma are at risk of being further excluded by undergraduate medical curricula. This paper enhances understanding of the challenges that are limiting Inclusion Health education. Most importantly, the paper presents solutions for how Inclusion Health can be incorporated into undergraduate medical teaching and assessment.

## 1 Introduction

The existence of a social gradient in health, where the lower the person's social position, the worse their health [1], is well recognised in undergraduate medical education, but there is evidence that medical students are not then learning how to address the healthcare needs of the most socially excluded people [2, 3]. In the United Kingdom and Ireland, the concept of Inclusion Health has evolved as an approach to bring together evidence and best practice to address the extreme health inequities experienced by the most marginalised groups. This typically encompasses people experiencing homelessness, problem substance use, Gypsy, Roma and Traveller communities, vulnerable migrants, sex workers, people in contact with the justice system and victims of modern slavery but can include other groups facing intersecting forms of exclusion [4]. Terms used internationally to describe similar populations can include ‘underserved’, ‘marginalised’ and ‘vulnerable’ [5]. A systematic review and meta‐analysis in 2018 described mortality ratios eight times higher than the average for men and nearly 12 times higher for women in Inclusion Health groups [6]. The extreme health inequities experienced are compounded by multiple barriers in accessing healthcare services [7, 8, 9, 10], including discriminatory and stigmatising behaviour by clinicians [11, 12].

Policy approaches to improving care in Inclusion Health have been gaining momentum with a national framework for action published in England in 2023 [13], in the same year the Scottish Government developed the Inclusion Health in General Practice programme [14] and an Inclusion Health framework for Ireland is currently under development. There is evidence that undergraduate medical education is failing to keep up with this progress; a study from 2015 reported that Inclusion Health is underdeveloped in undergraduate education in the United Kingdom and proposed that regulatory bodies should do more to make the topic explicit in their undergraduate curricula [2]. Recent research exploring medical students' experiences of Inclusion Health education reported that lectures and mainstream placements were leaving students feeling unprepared to work with socially excluded people [15]. Perhaps more concerning is international evidence that medical students develop increasingly negative attitudes towards Inclusion Health groups over the course of their training [16, 17].

*Inclusion Health is underdeveloped in undergraduate education in the United Kingdom*.

The influential Marmot Review urged that action to reduce the steepness of the social gradient in health must be universal but ‘with a scale and intensity that is proportionate to the level of disadvantage’ [1]. To truly address health inequities, should medical education apply this concept of ‘proportionate universalism’ [1] and increase the scale and intensity of education about care for the most disadvantaged groups? What is holding educators back from this work and what action could help address the problem?

### 1.1 Research Question

What are the challenges and opportunities in teaching Inclusion Health to medical students in the United Kingdom and Ireland?

## 2 Methods

### 2.1 Sampling and Recruitment

Purposive sampling was used to select participants with experience in teaching Inclusion Health at a range of medical schools in the United Kingdom and Ireland. Participants were initially recruited through emails to known contacts, with snowball sampling subsequently used to identify additional educators who may otherwise have been unknown to the research team. This combined approach was appropriate given the absence of a formal registry or clear visibility of Inclusion Health educators within medical schools. It enabled the intentional recruitment of participants able to give a detailed picture of the challenges and opportunities in teaching Inclusion Health to medical students, in accordance with the principal aims of the study.

### 2.2 Data Collection

Pilot testing of an interview guide, developed based on the research objectives and a previous scoping review of the literature [5], was undertaken before the initial interview. One‐to‐one semistructured interviews were conducted by the lead researcher (G.A.). Interviews were conducted remotely via Microsoft Teams over a 5‐month period from October 2023 to February 2024 (each lasting 50–75 min).

### 2.3 Data Analysis

The interview transcripts were analysed using reflexive thematic analysis, guided by the six‐phase process for data engagement, coding and theme development (Braun and Clarke [18, 19]). The transcripts were coded manually in Microsoft Word by G.A. with a subset of the data jointly coded by A.M.R. Themes were generated iteratively by organising codes around a ‘central organising concept’ interpreted from the data [18].

Due to the exploratory nature of the subject, an inductive approach was taken, with the coding and theme generation driven by the content rather than by existing theoretical constructs [20]. The analysis was underpinned by a critical realist ontology [20], which allowed us to centre the experiences of the participants, while also considering some of the cultural and social understandings that support their descriptions.

### 2.4 Reflexivity

The lead author acknowledges their position as a general practitioner (family physician) in Inclusion Health and medical educator. The influence of their positionality, beliefs and experiences in shaping the research process was examined through journaling and regular discussion with the supervisors and co‐authors [21].

## 3 Results

**Box 1. Key themes and subthemes.**

Five distinct themes and one subtheme were identified from the data:

3.1 ‘My goodness me, it's difficult to get that stuff in’; creating space for Inclusion Health in undergraduate curricula
3.2 ‘It's the human‐to‐human connection’; the importance of meaningful contact with people with lived experience
  3.2.1 Limited role for digital education
3.3 The impact of the hidden curriculum
3.4 ‘Assessment is the biggest hurdle’
3.5 Inclusion Health as a core competency for clinical practice

### 3.1 ‘My Goodness Me, It's Difficult to Get That Stuff In’; Creating Space for Inclusion Health in Undergraduate Curricula

Participants described undergraduate medical curricula that are overloaded and that prioritise biomedical sciences, while having substantial gaps relating to social justice issues:

They hardly have any teaching on deprivation, they get more teaching about Krebs cycle I think. (P13)

Other participants described that teaching Inclusion Health feels ‘countercultural’ in the current political climate, noting the prevalence of prejudiced views towards marginalised groups and a broader healthcare focus on profit. Several participants described either ‘fighting for little corners of space’ (P23) in curricula or using stealth to ‘sneak bits of Inclusion Health into teaching’ (P13). Inclusion Health was frequently described as being included through optional electives, student selected components or extracurricular activities that only benefit the small proportion of students opting into the learning:

What tends to happen is the people with a special interest join … one of the problems with that, of course, is that some of the people who probably would benefit from it most do not ever get exposed to it. (P12)

Participants also worried about the implicit message to students of only having Inclusion Health as optional components:

If only some students get it, then the message we are giving out is that this is not core. (P1)

Educators suggested that having explicit learning outcomes relating to Inclusion Health in national undergraduate medical education standards would help strengthen the case for allocating curriculum space to this topic.

### 3.2 ‘It's the Human‐to‐Human Connection’; the Importance of Meaningful Contact With People With Lived Experience

Overwhelmingly, educators in this study stressed the crucial role of attitudinal aspects in Inclusion Health. The importance of developing humanity, compassion, empathy and of being non‐judgemental was emphasised.

To cultivate the beneficial attitudes required, participants suggested the importance of meaningful student contact with people with lived experience:

It's the human‐to‐human connection. And to do that, they need to experience it and see it role modelled and have an opportunity to reflect on it. (P21)

Participants highlighted the benefits of learning from people with lived experience outside of the acute hospital environment where patients are more likely to be stressed or scared. Placements in Inclusion Health specialist services were deemed to be ideal locations for learning. However, the problem of large cohorts of students and limited Inclusion Health services was widely acknowledged. Participants suggested that innovative ideas for experiential learning in Inclusion Health could include joining street outreach teams, alcohol and drug recovery services, prison healthcare and third sector services such as day shelters and food banks.

Several participants had found other ways for students to learn with people with lived experience by involving them in small group work or lectures:

What you want is real stories. You want to have a real person who's been through it to tell you what it feels like to be ignored or to be treated badly by a doctor or a healthcare professional. (P13)

Involvement in central teaching was reported to be a potentially powerful learning experience, but also one that must be managed in a safe, trauma‐informed manner to ensure people are at an appropriate stage of recovery and are not put at risk of retraumatisation. Some educators shared experience of involving people with lived experience in the co‐production of teaching or the design of curricula. This was found to be an effective way of giving excluded patients a voice in medical education while being potentially less exposing than direct involvement in teaching.

#### 3.2.1 Limited Role for Digital Education

To improve access to experiential learning in Inclusion Health, the role of digital education has been considered. Several educators felt that relying on online learning risked undervaluing the interpersonal aspects that were thought to be crucial. There was also a concern that online learning could be used by medical schools as a ‘quick fix’ approach to Inclusion Health education. The use of digital education as part of a flipped classroom approach [22] to prepare students for placements or contact with people with lived experience was viewed more positively. The use of recorded patient narratives was seen by some as a way to challenge discrimination and exclusion; this was also seen as a potentially less intimidating way to involve people with lived experience. However, some educators worried that condensing a patient story into a short vignette risks missing important complexity and nuance. There is growing interest in live streaming consultations for medical education purposes and some participants thought this could help with the scalability of Inclusion Health experiences for medical students. There was concern about the use of such technology in consultations being potentially intrusive considering the vulnerability of many socially excluded patients. Other concerns included that students would miss out on the beneficial aspects of implicit learning expected from immersion in an Inclusion Health setting:

Nothing quite matches or substitutes for the experience of actually physically being in this setting, speaking to real patients about the issues that they have experienced, hearing their perspective, nothing comes close to that. (P21)

### 3.3 The Impact of the Hidden Curriculum

When Inclusion Health is being taught, participants described a dissonance between what is taught in the University setting and what the students are experiencing in mainstream placements. There were concerns that students are ‘learning poor behaviour from burnt out staff’ (P13), for example, ‘staff venting their frustration with patients’ (P11), blaming individual patients for their situation, not using interpreters when appropriate or observing ‘learned norms that self‐discharge is basically seen as a victory’ (P12).

Participants also noted implicit messages picked up by students about the perceived low prestige of working in Inclusion Health. Participants described concerns that Inclusion Health clinicians are seen as being ‘self‐sacrificing’ or that you might work in this field if you were unable to work in a higher status or higher earning role:

They're very low status patients and therefore it's very low status practice … So, when you could not make it in a rich middle‐class area, you have ended up in a homeless practice. (P7)

Ideas to address this negative implicit learning included increasing student exposure to positive role models in Inclusion Health, for example, with more placements in third sector organisations, in Inclusion Health specialist practices or more representation of Inclusion Health clinicians in medical education.

### 3.4 ‘Assessment Is the Biggest Hurdle’

There was concern that undergraduate medical assessment practices are not preparing students for the complexities and ambiguities of authentic, inclusive medical practice:

I think we have kind of slipped away from what it is to be a doctor in our assessment paradigm. (P16)

There was also concern from participants that important qualities for Inclusion Health care such as empathy and professionalism are difficult to assess in comparison with other topics:

It's easy to test anatomy, it's hard to test empathy. (P1)

Several participants felt that the move towards the Medical Licensing Exam in the United Kingdom will further exclude Inclusion Health from medical curricula:

I think a limitation of the of the MLA, it's a very reductionist way of looking at things which will serve some people for some things, but you know when it comes to this agenda of Inclusion Health, it doesn't serve it well. (P21)

Some participants viewed incorporating Inclusion Health into practical exams such as OSCEs to be a more appropriate way to assess the topic than multiple choice questions. Suggestions also included involving people with lived experience in the design of assessment to encourage authenticity and avoid stereotyping. Project work, the use of reflection and work‐based assessments were also proposed as potentially more realistic forms of assessment on Inclusion Health.

### 3.5 Inclusion Health as a Core Competency for Clinical Practice

Participants highlighted that resident doctors care for patients experiencing marginalisation, from the start of their role, and that medical education should prepare students to manage the complexity of care for all patients. There was agreement that Inclusion Health should be a core competency, integrated throughout the curriculum, rather than considered a niche subject to teach in isolation:

This is a really important area that's cross‐cutting, so it filters into antenatal care, oncology care, it filters into everything. (P9)

Rather than displacing other content, participants suggested that Inclusion Health could be woven into existing teaching across a range of specialities within undergraduate medical education.

One participant suggested an approach based on the seminal Marmot Review [1] to argue for increased Inclusion Health education:

I think the most strategic approach would be to listen to the population health scientists and say … where is the greatest burden of illness? Well, let us perhaps recognize that the burden of illness is higher for these people, and so let us have some ‘proportionate universalism’ in medical education. (P15)

Participants described how Inclusion Health exemplifies fundamental values such as empathy, advocacy, cultural humility and trauma‐informed care, and if we can equip students to get it right for the most excluded patients, then we will improve care all round:

I think it needs a sort of rethink in terms of who are we teaching our students to look after. It does have to be everybody. And perhaps if we were able to meet the needs of the most excluded people, then we would be able to look after everybody better. (P2)

## 4 Discussion

The need for medical schools to better prepare students to care for the most socially excluded patients was a view widely expressed by participants in this study. There seems to have been limited change since a study published on Inclusion Health education in the United Kingdom 10 years ago, which reported that the topic is underdeveloped in undergraduate healthcare curricula [2].

This study has explored the challenges that are limiting Inclusion Health education, as well as possible opportunities for change.

### 4.1 Challenges

Participants suggested that when Inclusion Health teaching is available, it is often limited to optional components or extracurricular activities. This aligns with findings from a scoping review of Inclusion Health education worldwide [5]. The current study expands on these findings, suggesting that educators keen to teach Inclusion Health are having to add the content ‘by stealth’ or ‘fight for space’ due to limited curricula time, the prioritisation of biomedical knowledge and the lack of explicit learning objectives relating to Inclusion Health in national undergraduate medical education curriculum guidance [23, 24].

Educators in this study were also concerned about students observing negative behaviour during mainstream placements. The risks of medical students witnessing poor care, stigma and discrimination towards Inclusion Health groups during clinical placements have been described in multiple papers [5, 15, 25], this research has identified possible solutions which are described in the next section. Inclusion Health specialist services were seen by participants in this study as ideal locations for students to experience positive clinical role models working with socially excluded patients, but the problem of limited capacity was acknowledged.

Current assessment paradigms were viewed as a further significant barrier to medical student engagement with Inclusion Health. This conforms with prior research which has highlighted the difficulty of engaging students in Inclusion Health if it is not perceived to have significant weight in examinations [2, 26].

Opportunities to address these key challenges have been drawn from the findings of this research.

### 4.2 Opportunities

#### 4.2.1 Diverse Experiential Learning

Medical schools across the United Kingdom and internationally are facing placement capacity challenges. By exploring more diverse opportunities for experiential learning in Inclusion Health services and within third sector organisations, institutions could expand placement capacity while educating students about the needs of people experiencing social exclusion.

#### 4.2.2 Lived Experience Involvement

Human‐to‐human connection was seen as crucial in Inclusion Health education. In addition to opportunities for connection during diverse placements, educators worked with people with lived experience in central teaching and in the codesign of Inclusion Health education. Positive impacts of involving service users in the co‐production of healthcare professions' education have been shown in recent research [27], involving people with lived experience could also be an effective way of giving marginalised patients a voice in medical education.

#### 4.2.3 Digital Potential: Incorporating Patient Narratives

A further option for incorporating the voice of socially excluded patients is through recorded patient narratives. A recent systematic review has shown that narrative medicine can be an effective pedagogic tool [28]. Educators in our study felt that this should not replace human‐to‐human connection but could be a useful adjunct to prepare students for placements or connection with people with lived experience.

#### 4.2.4 Innovative Assessment

As countries across the world transition towards licensing exams, like the MLA introduced into the United Kingdom in 2024–2025, the aim for standardisation risks reducing education on complexity, multimorbidity and uncertainty [29], all of which are key aspects of Inclusion Health. Our study highlights possible solutions such as co‐producing assessments with people with lived experience of social exclusion, project work, reflection and work‐placed based assessments in Inclusion Health, aiming to ensure adequate preparation for the complexity of real‐life practice.

#### 4.2.5 Explicit Learning Outcomes

Inclusion Health should be considered a core competency that can be woven into existing content across a variety of specialities, providing contextual learning opportunities. Just as safeguarding has recently become a core competency in UK undergraduate medicine [24], the development of explicit national learning outcomes would empower educators to integrate Inclusion Health into their curricula.

*The development of explicit national learning outcomes would empower educators to integrate Inclusion Health into their curricula*.

### 4.3 Recommendations for Future Research

Educators in this study expressed the need for detailed guidance on content and pedagogy in Inclusion Health education. The involvement of people with lived experience and students in shaping future research and guidance is crucial.

### 4.4 Strengths and Limitations

Participants in this study were recruited from 19 different medical schools across the United Kingdom and Ireland and from backgrounds including general practice, secondary care, public health and the social sciences; this allowed for diversity within the boundaries of the sample, enabling a range of factors associated with teaching Inclusion Health in undergraduate medical education to be explored. Due to the nature of the research question and purposive sampling to recruit educators with experience of teaching related to Inclusion Health, the educators included in this study were generally proponents of Inclusion Health. Examining the perspectives of educators who are indifferent or opposed to the integration of Inclusion Health into undergraduate medical curricula may provide important insights. Although beyond the scope of this study, such inquiry could be a valuable direction for future research.

## 5 Conclusion

Medical schools should prepare students to care for the full diversity of people they will encounter from their first day of work in healthcare, including people from socially excluded groups. This paper outlines what can be done to achieve this from the perspective of Inclusion Health educators in the United Kingdom and Ireland. Although conducted in this context, the findings have clear international relevance: challenges such as curriculum crowding, the hidden curriculum and barriers to meaningful assessment are common across medical education when teaching about marginalised groups. While the specific populations encompassed by ‘Inclusion Health’ differ globally, underlying issues of exclusion and inequity are universal. The themes and recommendations identified here can therefore inform curricular reform internationally and help medical schools shape a future workforce capable of addressing extreme health inequities.

Further research exploring the views of people with lived experience and medical students is needed to build on this work and inform future guidance.

*Medical schools should prepare students to care for the full diversity of people they will encounter from their first day of work in healthcare, including people from socially excluded groups.*

## Author Contributions

**Gemma Ashwell:** conceptualization, data curation, formal analysis, investigation, methodology, project administration, supervision, validation, visualization, writing – original draft, writing – review and editing. **Amy M. Russell:** conceptualization, methodology, supervision, validation, writing – review and editing. **Lindsey Pope:** conceptualization, methodology, supervision, validation, writing – review and editing. **Andrea Williamson:** conceptualization, methodology, supervision, validation, writing – review and editing.

## Funding

The authors received no specific funding for this work.

## Ethics Statement

The study was approved by the University of Leeds Research Ethics Committee reference code: MREC 22‐085.

## Consent

Participants all gave informed consent in writing to take part in this research study and for the data to be submitted for publication.

## Conflicts of Interest

The authors declare no conflicts of interest.

## Data Availability

The data that support the findings of this study are available on request from the corresponding author. The data are not publicly available due to privacy or ethical restrictions.
